# Supplementary material for: Preparation and Characterization of Screen-Printed Cu2S/PEDOT:PSS Hybrid Films for Flexible Thermoelectric Power Generator
Source: Nanomaterials (Basel). 2022 Jul 15;12(14):2430. doi: 10.3390/nano12142430 (PMC9324872; doi:10.3390/nano12142430)
Supplement: Supplementary file 1 [file nanomaterials-12-02430-s001.zip › nanomaterials-1783487-supplementary.pdf]

nanomaterials

MDPI

Supplementary Materials

Preparation and Characterization of Screen-Printed Cu_2S/PEDOT:PSS Hybrid Films for Flexible Thermoelectric Power Generator

Junmei Zhao [1], Xiaolong Zhao [1], Rui Guo [2], Yaxin Zhao [2], Chenyu Yang [1,2], Liping Zhang [1], Dan Liu [1,2,*] and Yifeng Ren [1,*]

1 School of Electrical and Control Engineering, North University of China, Taiyuan 030051, China; zhaojunmei@nuc.edu.cn (J.Z.); zhaoxiaolong@nuc.edu.cn (X.Z.); yangwangcgsa@163.com (C.Y.); zhangliping@nuc.edu.cn (L.Z.)
2 Key Laboratory of Instrumentation Science & Dynamic Measurement, Ministry of Education, North University of China, Taiyuan 030051, China; 18406589626@163.com (R.G.); zhaoyaxinnuc@163.com (Y.Z.)
* Correspondence: liudan235@nuc.edu.cn (D.L.); renyifeng@nuc.edu.cn (Y.R.)

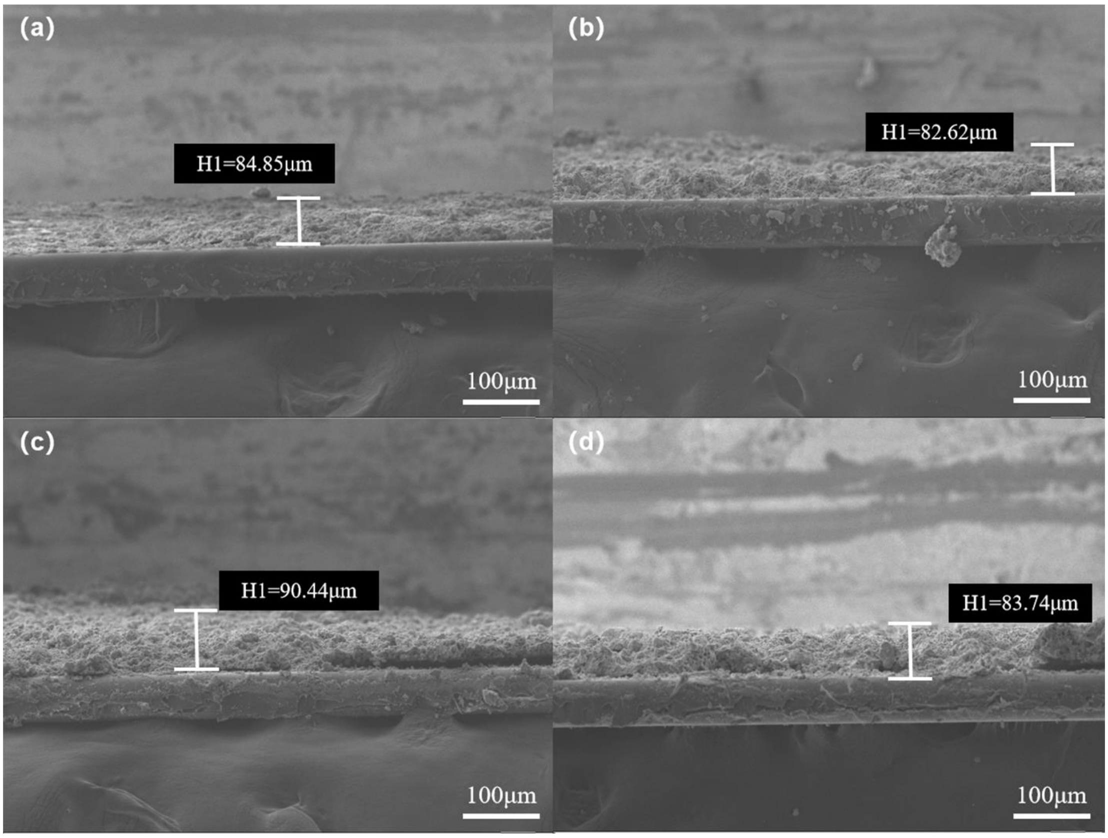

Figure S1. The cross-sections of (**a**) P1.1, (**b**) P1.2, (**c**) P1.3, and (**d**) P1.4 films.

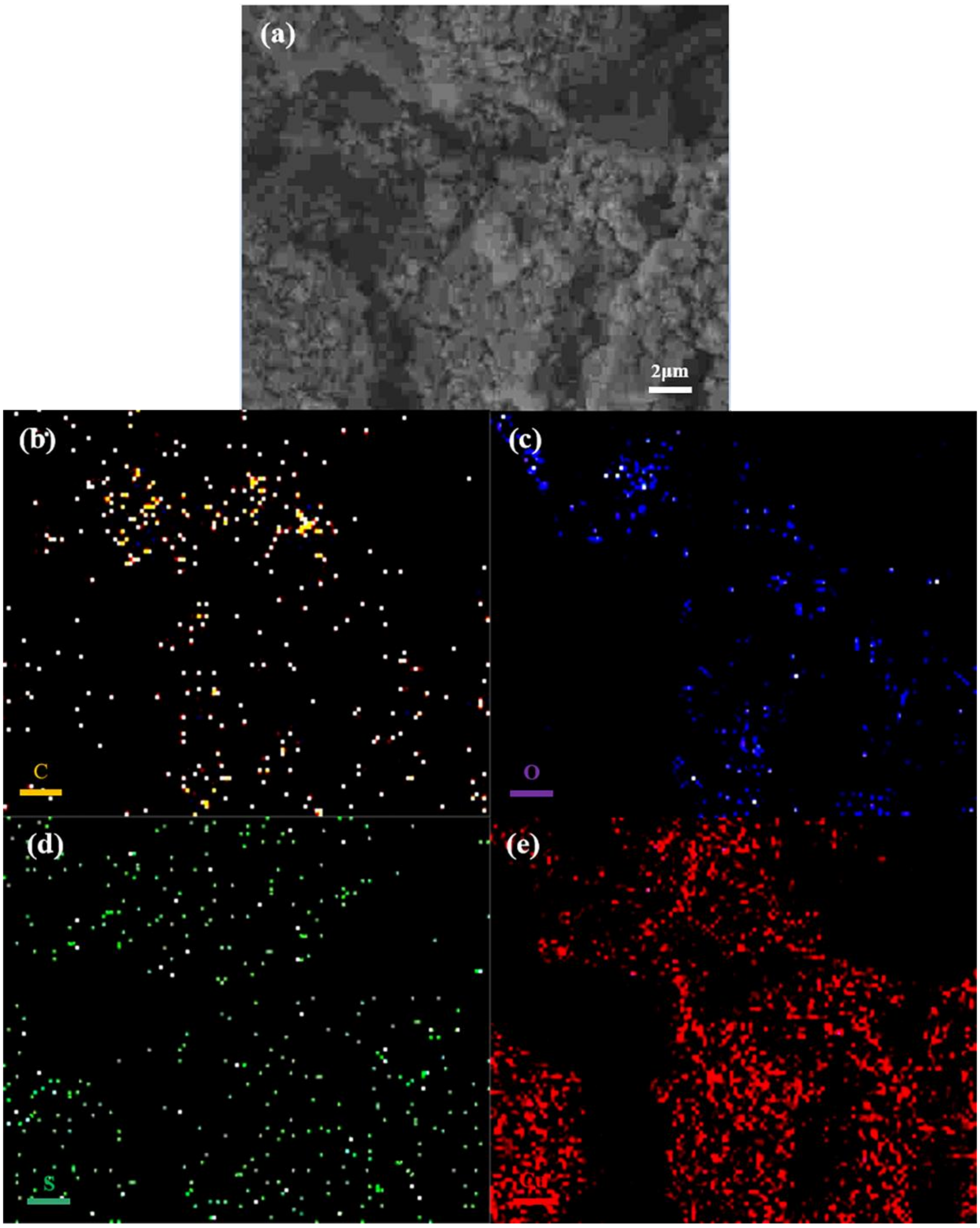

Figure S2. Element mapping diagram of P1.2 film, (**a**) SEM diagram of P1.2 film, (**b**) C element, (**c**) O element, (**d**) S element, (**e**) Cu element.

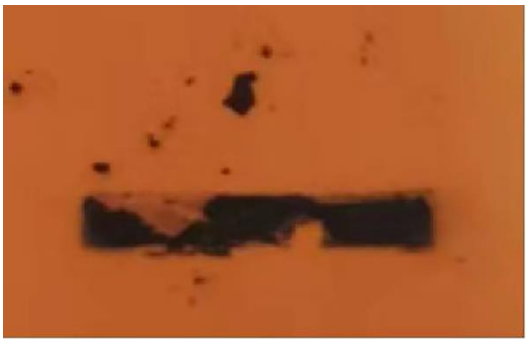

Figure S3. The image of the P1.1 film module no longer adheres to the PI substrate.
